# Supplementary material for: Somatic regulation of female germ cell regeneration and development in planarians
Source: Cell Rep. Author manuscript; Available in PMC 2022 Apr 9. (PMC8994625; doi:10.1016/j.celrep.2022.110525)
Supplement: 1 [file NIHMS1789672-supplement-1.pdf]

Cell Reports, Volume *38*

Supplemental information

Somatic regulation of female germ cell regeneration and development in planarians

Umair W. Khan and Phillip A. Newmark

Cell Reports, Volume *38*

Supplemental information

Somatic regulation of female germ cell regeneration and development in planarians

Umair W. Khan and Phillip A. Newmark

A

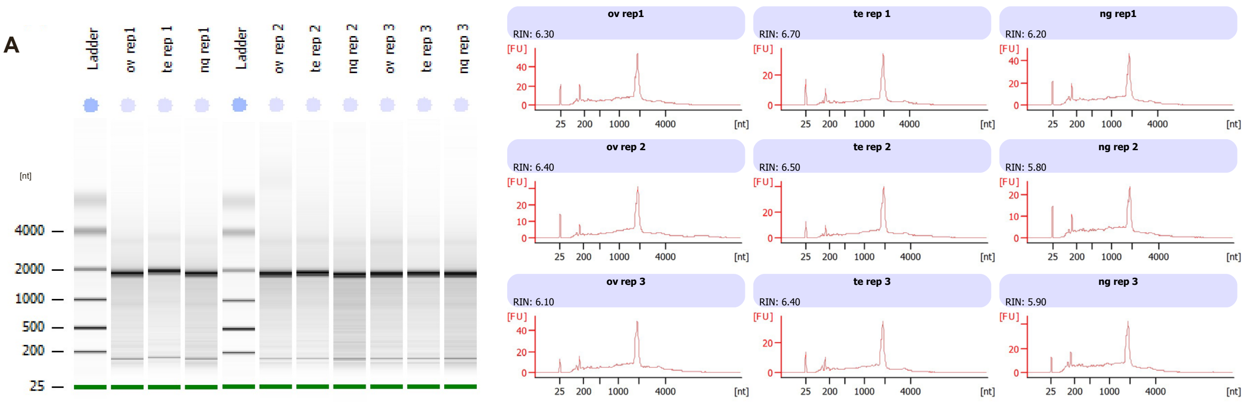

B

Sample	RNA yield (ng)
ov-rep1	37.44
ov-rep2	101.92
ov-rep3	76.44
te-rep1	28.16
te-rep2	87.62
te-rep3	57.72
ng-rep1	29.04
ng-rep2	74.62
ng-rep3	75.48

C

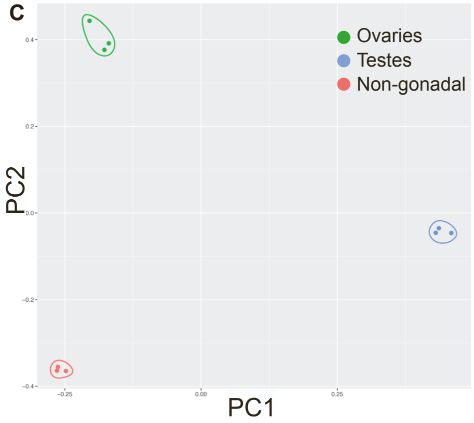

D

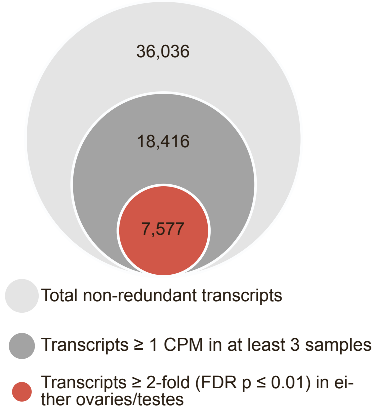

Figure S1. LCM-RNA-seq approach to generate gonadal transcriptomes, Related to Figure 1.
(A) Bioanalyzer analysis of RNA integrity recovered from LCM-excised tissue. (B) RNA yields from LCM-dissected tissues (ov- ovary, te- testis, ng- non-gonadal, rep- replicate) (C) Principle component analysis of LCM-RNA-seq samples. (D) LCM-RNA-seq results: 7,577 genes (≥ 2-fold; FDR p≤ 0.01) are preferentially expressed in ovaries or testes compared to non-gonadal tissue.

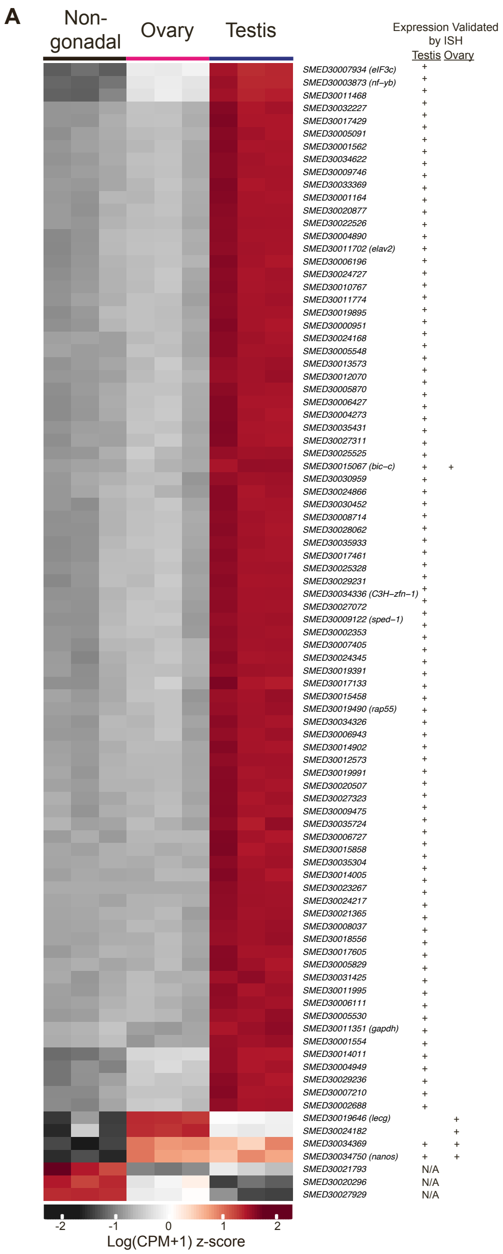

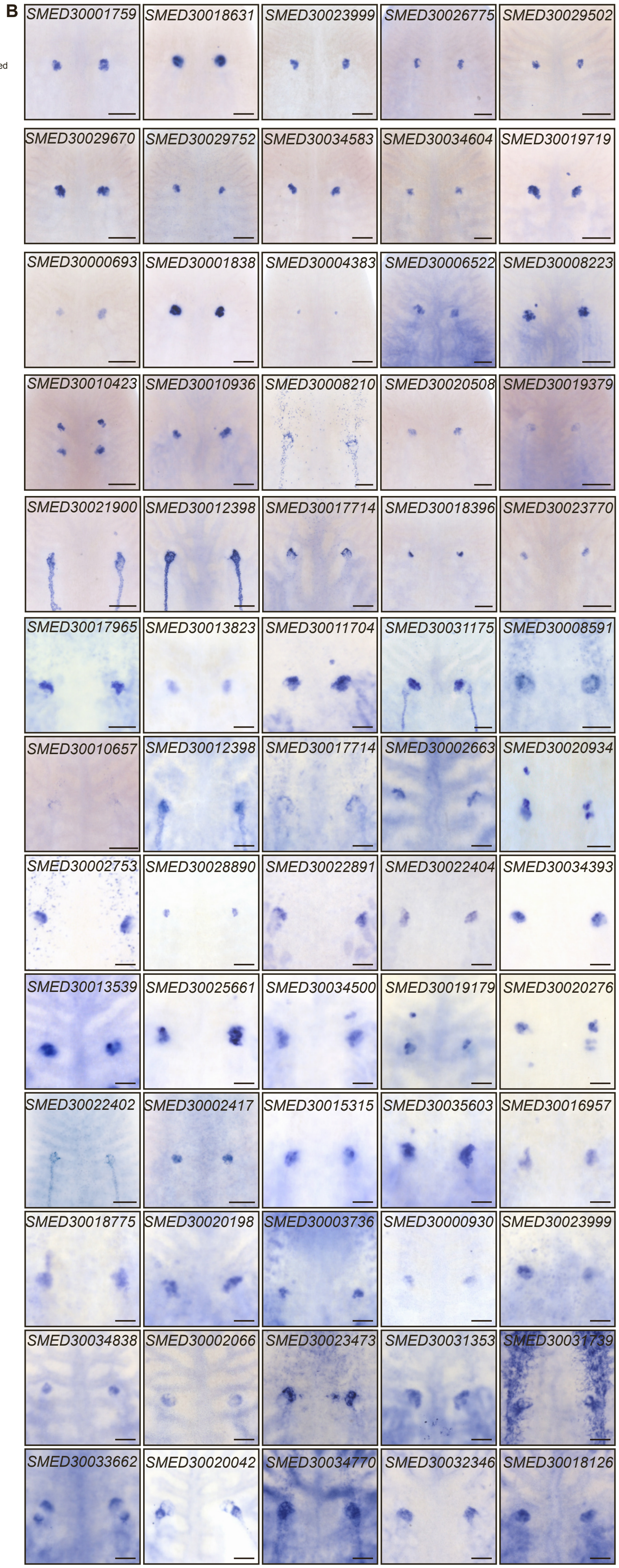

Figure S2. Validation of LCM-RNA-seq-generated gonadal transcriptomes, Related to Figure 1.
(A) Heatmap of 89 gonadal-enriched genes previously identified and validated (Wang et al. 2010). ISH results from Wang et al. study summarized in the table to the right. (B) Representative WISH for additional ovary-enriched candidate genes. Scale bars: 200 μm.

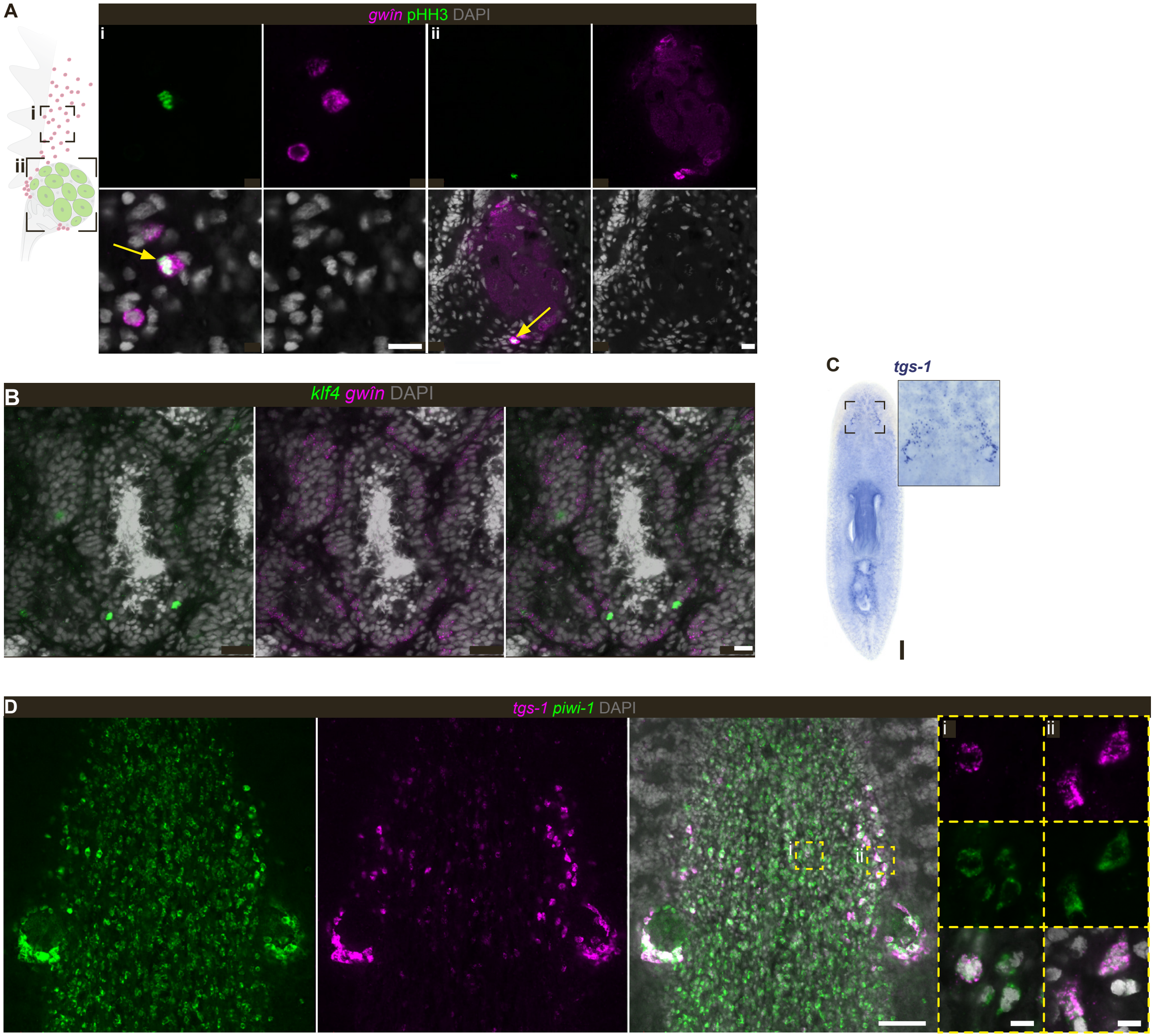

Figure S3. FGPs are a proliferative, extra-ovarian cell population, defined by coexpression of *klf4* and *gwîn*, Related to Figure 2.
(A) FISH to detect *gwîn* and anti-phospho histone H3 (pHH3) indicating proliferative activity of FGPs. (B) FISH to detect *gwîn* expression in testes. (C) WISH to detect *tgs-1* reveals enriched expression in cells around the margin of the ovary and anterior to the ovary. *tgs-1* expression is not detected in the testes. (D) FISH to detect *tgs-1* and *piwi-1*. *tgs-1* marks a pluripotent subset of neoblasts (i) and is enriched in FGPs (ii). Scale bars: (A,B) 20 µm; (C) 500 µm; (D) 100 µm; insets: 10 µm.

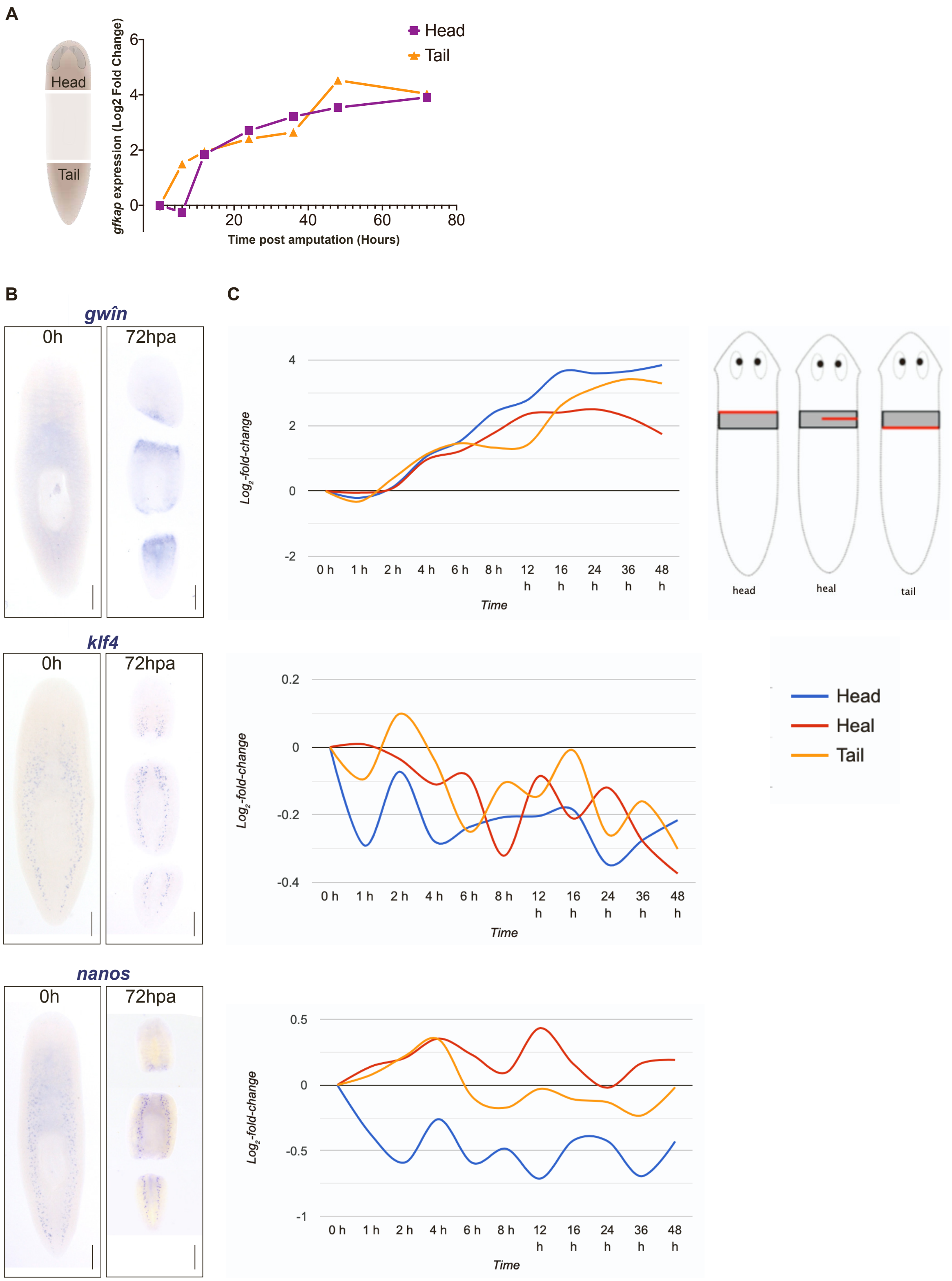

Figure S4. Expression of germ cell markers after wounding, Related to Figure 2.
(A) Expression of *gwîn* in head and tail fragments after amputation. Regeneration time course data (Kao et al., 2013) for *gwîn* retrieved from Planmine (http://planmine.mpi-cbg.de/)(Rozanski et al., 2019). (B) WISH for *gwîn*, *nanos* and *klf4* at 0 hours and 72 hours post-amputation (hpa) shows that only *gwîn* has detectable upregulation at wound sites after injury. (C) Expression of *gwîn*, *klf4* and *nanos* during a regeneration time course retrieved from Planmine. Red: injury site; grey box: tissue analyzed.

Figure S5

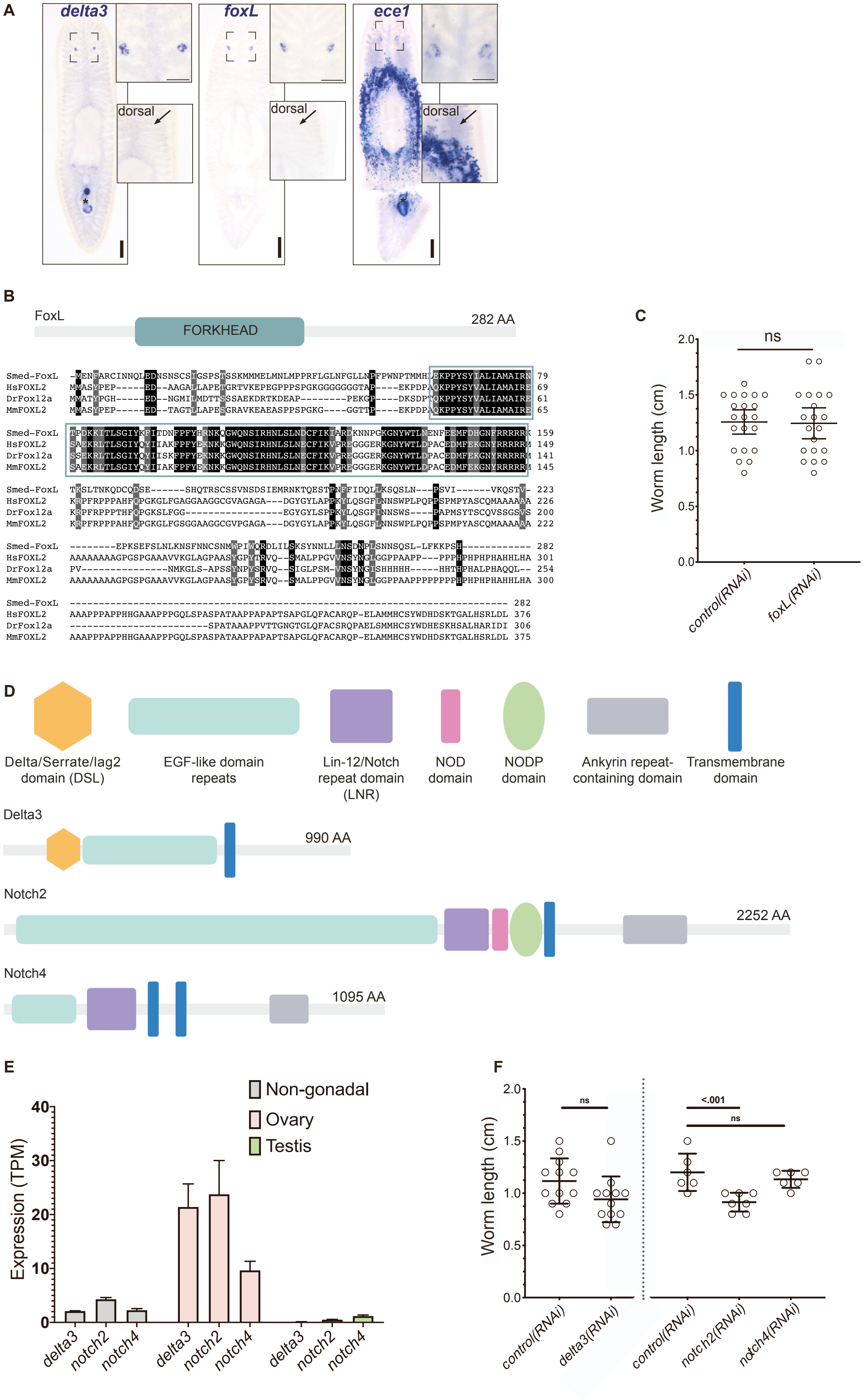
A
delta3
foxL
ece1
dorsal
dorsal
dorsal
B
FoxL
FORKHEAD
282 AA
Smed-FoxL
HsFOXL2
DrFoxl2a
MmFOXL2
C
ns
2.0
1.5
1.0
0.5
0.0
Worm length (cm)
control(RNAi)
foxL(RNAi)
D
Delta/Serrate/lag2 domain (DSL)
EGF-like domain repeats
Lin-12/Notch repeat domain (LNR)
NOD domain
NODP domain
Ankyrin repeat-containing domain
Transmembrane domain
Delta3
990 AA
Notch2
2252 AA
Notch4
1095 AA
E
Non-gonadal
Ovary
Testis
Expression (TPM)
40
30
20
10
0
delta3
notch2
notch4
delta3
notch2
notch4
delta3
notch2
notch4
F
ns
<.001
ns
2.0
1.5
1.0
0.5
0.0
Worm length (cm)
control(RNAi)
delta3(RNAi)
control(RNAi)
notch2(RNAi)
notch4(RNAi)

Figure S5. Analysis of genes expressed in ovarian support cells, Related to Figures 3 and 4.

(A) WISH for *delta3, foxL, and ece1* indicating expression in the ovary. Dorsal view shows that none of these genes have detectable expression in the testes (arrows pointing to the region where testes are found). *ece1* and *delta3* are detected in the penis papilla (asterisks), while *ece1* is highly expressed in the secretory cells around the pharynx as well. (B) Sequence alignment of Smed-FoxL with vertebrate FoxL2 proteins from Humans (Hs), Mouse (Mm), and Zebrafish (Dr). Black and gray shading indicate residues that are identical or conserved, respectively, among all four species. (C) Measurement of *control(RNAi)* and *foxL(RNAi)* worm length at the end of RNAi regeneration assay (ns: not significant; mean with 95% CI). (D) Domain architecture analysis of Smed-Delta3, -Notch2, and Notch4 using SMART and Interpro protein domain analysis tools. Smed-Delta3 contains a N-terminal Delta/Serrate/Lag2 (DSL) domain and EGF (epidermal growth factor) motifs. (E) *delta3*, *notch2*, and *notch4* mRNA expression levels (TPM- Transcripts per million) in the ovary, testis, and non-gonadal transcriptomes. (F) Measurement of *control(RNAi)*, *delta3(RNAi)*, *notch2(RNAi)*, and *notch4(RNAi)* worm length at the end of RNAi regeneration assay (ns: not significant).

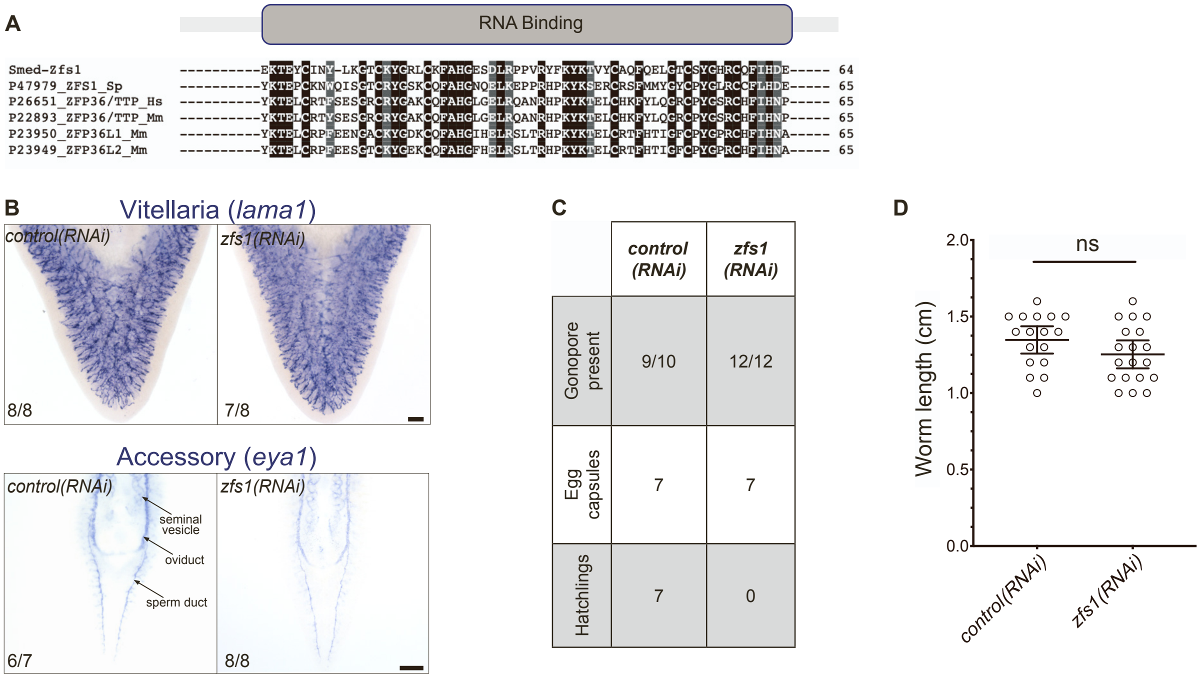

Figure S6. Female-specific role of *zfs1* in germ cell regeneration, Related to Figure 5.
(A) Sequence alignment of tandem zinc finger (C3H1) RNA-binding domain of Smed-Zfs1 with homologs from Yeast (Sp), Humans (Hs), and Mouse (Mm). Black and gray shading indicate residues that are identical or conserved. (B) WISH for *lama1* (vitellaria), and *eya1* (accessory reproductive organs) in control and *zfs1(RNAi)* worms (tails regenerating a head). (C) Presence of gonopore, egg-laying, and egg-hatching observed for *control (RNAi)* and *zfs1(RNAi)* regenerated worms. (D) Measurement of *control(RNAi)* and *zfs1(RNAi)* worm lengths at the end of RNAi regeneration assay (ns: not significant). Scale bars: (B) 200 μm.

Figure S7

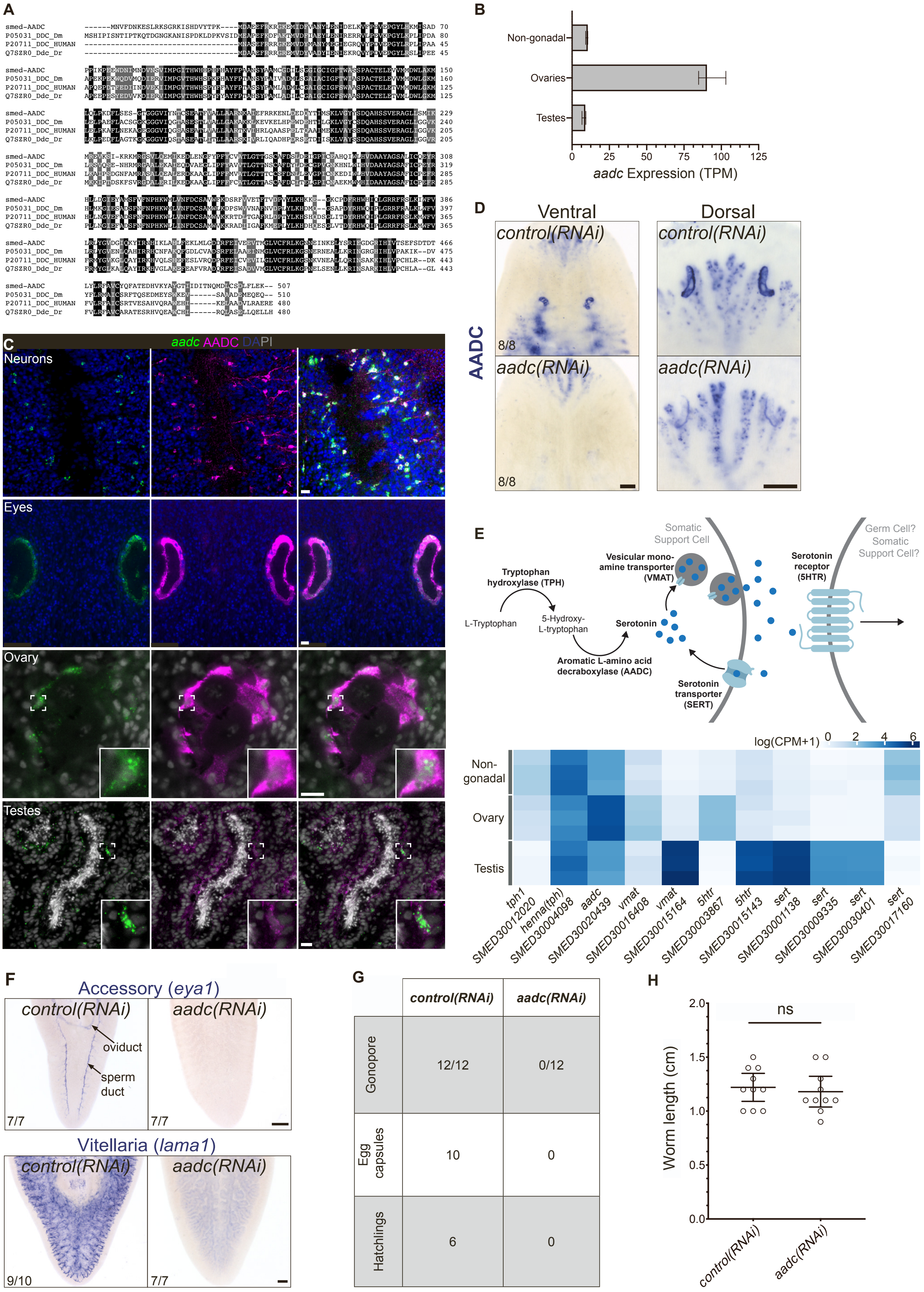

	control(RNAi)	aadc(RNAi)
Gonopore	12/12	0/12
Egg capsules	10	0
Hatchlings	6	0

Figure S7. Roles of AADC in germ cell and reproductive development, Related to Figure 6.

(A) Sequence alignment of Smed-AADC with AADC proteins from Humans (Hs), Mouse (Mm), and Zebrafish (Dr). Black and gray shading indicate residues that are identical or conserved, respectively, among all four species.(B) *aadc* expression levels (TPM) in the ovary, testis, and non-gonadal transcriptomes. (C) Expression of *aadc* mRNA and AADC protein in the indicated cell types and organs. (D) Labeling for AADC protein after *aadc* knockdown ablates labeling in the ovaries, eyes, and neurons, verifying the specificity of the AADC antibody staining in these organs. Non-specific labeling of gut branches can be seen in the background. (E) Schematic of canonical serotonin synthesis and signaling pathway in gonadal cells (Left). Normalized expression levels (LogCPM (counts per million)) of serotonin signaling pathway components in planarian gonads and non-gonadal tissues from the LCM-generated transcriptomes. (F) WISH for *lama1* (vitellaria), and *eya1* (accessory reproductive organs) in *control(RNAi)* and *aadc(RNAi)* regenerated worms (tails regenerating a head). (G) Presence of gonopore, egg-laying, and egg-hatching observed for *control(RNAi)* and *aadc(RNAi)* regenerated worms (tails regenerating a head). (H) Measurement of *control(RNAi)* and *aadc(RNAi)* worm lengths at the end of RNAi regeneration assay (ns: not significant). Scale bars: (C) 20 μm; (D, F) 200 μm.
